# Olfactory Neuroblastoma: Re-Evaluating the Paradigm of Intracranial Extension and Cyst Formation

**DOI:** 10.3390/diagnostics12030614

**Published:** 2022-03-01

**Authors:** Rebecca A. Dumont, Miguel Fernando Palma Diaz, William Hsu, Ali R. Sepahdari

**Affiliations:** 1 Neuroradiology Section, Department of Radiological Sciences, David Geffen School of Medicine, University of California, Los Angeles, CA 90095, USA; 2 Diagnostic Department, Division of Radiology, University Hospitals of Geneva, University of Geneva, 1205 Geneva, Switzerland; 3 Department of Pathology and Laboratory Medicine, David Geffen School of Medicine, University of California, Los Angeles, CA 90095, USA; miguel.f.palma-diaz@kp.org; 4 Medical & Imaging Informatics, Department of Radiological Sciences, David Geffen School of Medicine, University of California, Los Angeles, CA 90095, USA; willhsu@ucla.edu; 5 Diagnostic Radiology, Scripps Clinic Torrey Pines, La Jolla, CA 92037, USA; ali.sepahdari@gmail.com

**Keywords:** olfactory neuroblastoma, esthesioneuroblastoma, MRI, radiology–pathology correlation

## Abstract

The purpose of the current study was to assess the prevalence of cyst formation at the brain-tumor interface in olfactory neuroblastoma. We used the UCLA patient-based Pathology and Radiology Head and Neck Database (UPP&R HAND) to identify the largest patient cohort reported to date with imaging and pathology data. Eighteen of thirty-one patients (58.1%) had evidence of intracranial extension on MRI, while four (22.0%) demonstrated cyst formation at the brain–tumor interface. The extent of intracranial extension was by far the strongest predictor for intracranial cyst formation, regardless of Hyams tumor grade, using a binary logistics regression model (*p* = 0.002) and ROC curve analysis (AUC 94.6%). Cyst formation at the brain-tumor interface was an uncommon imaging finding, and tends to occur with a larger component of intracranial tumor extension.

## 1. Introduction

Olfactory neuroblastoma, also known as esthesioneuroblastoma, is an uncommon malignant neuroectodermal neoplasm that typically arises within the superior nasal cavity in proximity to the cribriform plate [1]. Classic teaching regarding the imaging characteristics of these lesions includes the formation of cysts at the margin of the brain-tumor interface [2,3], however the true prevalence of this finding is controversial. Using the cross-sectional UCLA patient-based Pathology and Radiology Head and Neck Database (UPP&R HAND) to identify a patient cohort with both imaging and corresponding pathology data, we evaluated the prevalence of intracranial extension in olfactory neuroblastoma and the corresponding prevalence of cysts at the brain-tumor interface. Additionally, we hypothesized that cyst formation is associated with larger intracranial tumor size and higher histological tumor grade.

## 2. Methods

### 2.1. Patients

Patients with olfactory neuroblastoma were identified from the UCLA UPP&R HAND Database, which comprises data on patient epidemiology, pathology, imaging and outcome of all consecutive patients seen at the UCLA Johnson Comprehensive Cancer Center between 2000 and 2013 with head and neck cancers. A cross-sectional study design was used to assess the prevalence of intracranial extension in olfactory neuroblastoma and the formation of cysts at the brain tumor interface. The study was approved by the UCLA institutional review board.

### 2.2. Imaging and Analysis

MR imaging of the head and neck was performed at UCLA on both 1.5 and 3 tesla MR units (Siemens, General Electric, Munich, Germany) and included coronal and axial T1 (TR 500–600 ms, TE 8–16 ms), coronal and axial FSE T2 (TR 3000–4000 ms, TE 102–108 ms), and axial and either coronal or sagittal post-gadolinium T1 (gadopentate dimeglumine (Magnevist, Schering, Berlin, Germany) 0.1 mmol/L per kilogram of body weight) with fat saturation.

Each case was assessed in duplicate to ensure accuracy by two neuroradiologists (R.A.D., A.R.S.) blinded to all clinical and histopathological data. Cases were reviewed independently and by consensus in the event of discordance. MR images were analyzed for the presence or absence of intracranial extension and cyst formation, as well as measurement of the maximum craniocaudal intracranial tumor dimension as seen on coronal or sagittal post-contrast T1-weighted images. Intracranial extension was defined by clear imaging evidence of tumor extension through the cribiform plate. Peritumoral cyst formation was defined by the presence of at least one focal collection of intracranial T2 hyperintensity located at the tumor-brain interface. The largest craniocaudal intracranial tumor dimension was measured using the standard GE PACS measuring tool function.

### 2.3. Specimen Analysis

Pathologic specimens were obtained by either biopsy or surgical resection, and were evaluated histologically and immunohistologically, and scored for Hyams tumor grade [4], by the UCLA Department of Pathology and Laboratory Medicine. All pathological specimens were assessed blinded to clinical data and imaging results. Reasons for no score included the presence of only microfoci of tumor on sampling, or pathology obtained from an outside institution that was unable to be reliably scored. Hyams grade I and II were considered low grade, while grades III and IV were considered high grade, as described in the literature [4].

Histologic diagnosis of olfactory neuroblastoma included standard criteria as accepted in the current Pathology literature (ref), such as the presence of nests and lobules of monotonous tumor cells with round nuclei, indistinct nucleoli and scanty cytoplasm in association with a vascular-rich to hyalinized stroma as well as the presence of Homer Wright pseudorosettes in low grade lesions (neoplastic cells palisading around a central zone of fibrillar neural matrix) or Flexner–Wintersteiner rosettes in higher grade lesions (palisading tumor cells surrounding a true central lumen). Immunostaining was used to confirm histological findings, and included diffuse synaptophysin staining of tumor cells and S100 staining in sustentacular cells at the periphery of tumor cell nests [5].

### 2.4. Statistical Analysis

All statistical analyses were performed using SPSS (v 25.0 IBM SPSS Statistics for Mac, IBM Corp., Armonk, NY, USA) or Graphpad (v 7.0 for Mac; GraphPad Software, San Diego, CA, USA). Two-sided *p* values of ≤ 0.05 were considered statistically significant. For all statistical analyses, tumors with grades I and II were grouped into “low grade” and tumors with grades III and IV were grouped into “high grade” categories according to Hyams grading, as is considered standard practice. Fisher’s exact tests using a 2 × 2 contingency table were performed to evaluate the relationships between tumor grade (high versus low) and intracranial extension (present versus absent), tumor grade (high versus low) and cysts in tumors with intracranial extension (present versus absent), survival (alive versus dead) and intracranial tumor extension (present versus absent), and survival (alive versus dead) and tumor grade (high versus low).

A binary logistic regression model was performed with “cyst formation” as the dependent variable and “intracranial tumor size” as an independent variable. A Receiver Operating Characteristic (ROC) curve was generated using “intracranial tumor size” as a test variable and “cyst formation” as the state variable.

## 3. Results

**Figure 1:**
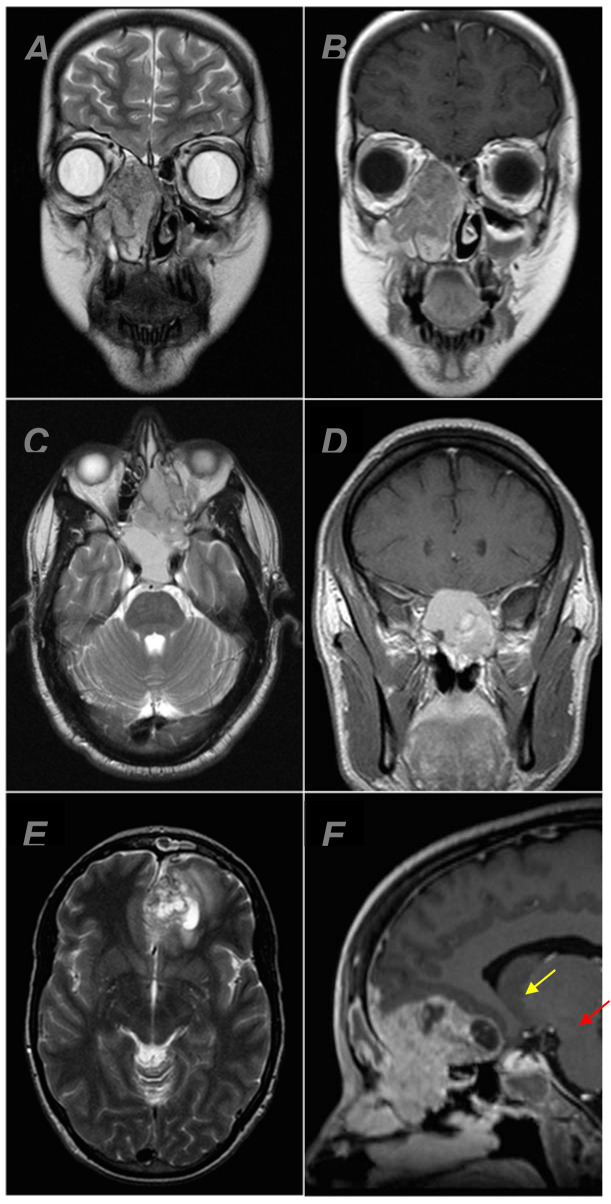
MRI examples of olfactory neuroblastoma centered in the nasal cavity. Coronal T2 (**A**) and T1 + C (**B**) showing an olfactory neuroblastoma without intracranial extension. Axial T2 (**C**) and coronal T1 + C (**D**) showing an olfactory neuroblastoma with intracranial extension and no peritumoral cyst formation. Axial T2 (**E**) and sagittal T1 + C (**F**) showing an olfactory neuroblastoma with intracranial extension and at least one focus of peritumoral cyst formation (red arrow). The area delimited by the yellow arrow was favored to represent necrosis, which was confirmed on pathology.

A total of 31 patients with olfactory neuroblastoma (13 females, 18 males, median age: 55 years, range: 12–79 years) were identified in UPP&R HAND between 2000 and 2013 who had both pertinent imaging and pathology data (Table 1). Eighteen patients (58.1%) had evidence of intracranial extension. Four of these eighteen patients (22.0%) demonstrated cyst formation at the brain–tumor interface, all of which demonstrated peripheral enhancement (Figure 1).

**Figure 2:**
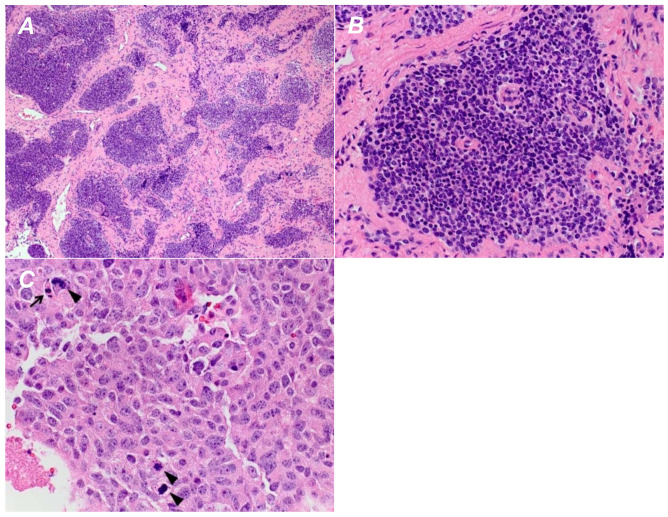
Low-power view of an olfactory neuroblastoma displaying a well-defined lobular growth pattern (**A**). On higher magnification, each lobule is composed of a uniform population of cells with minimal pleomorphism (**B**). No increased mitotic activity or evidence of necrosis is seen. These features are consistent with a Hyams grade 1 olfactory neuroblastoma. (**C**) A case of olfactory neuroblastoma with prominent pleomorphism and several mitotic figures (arrowheads). Scattered apoptotic cells are also identified (arrow). These features are those of Hyams grade 4.

**Figure 3:**
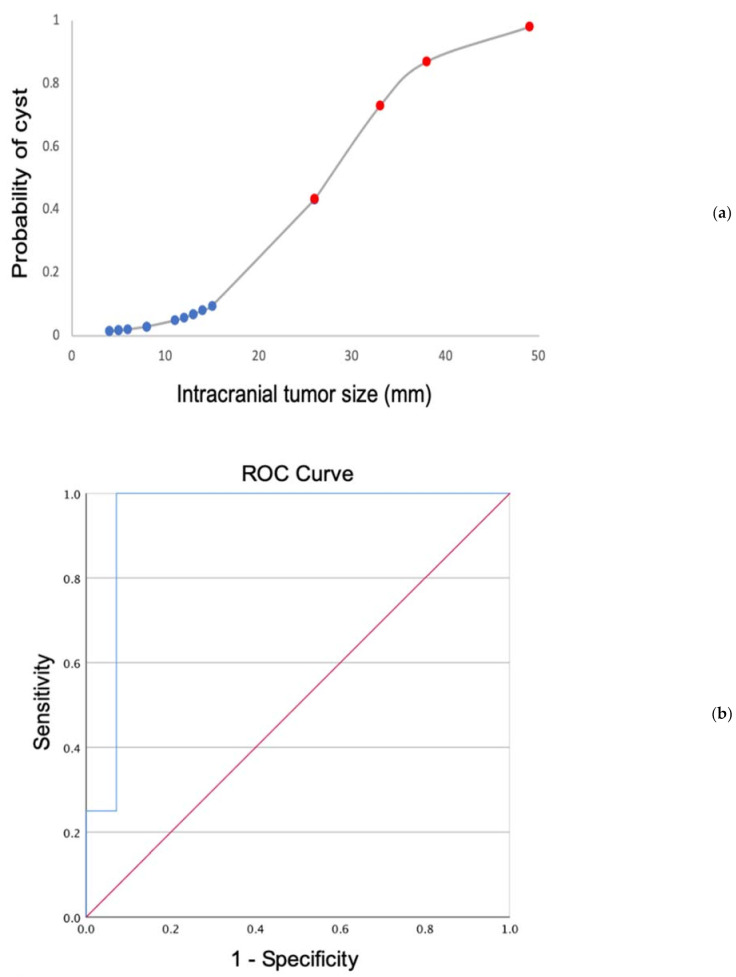
(**a**) Binary fitted line plot displaying the logistics regression equation using the 18 cases with intracranial extension. Red dots indicate tumor cases with cysts, while blue dots indicate those without cyst formation. (**b**) ROC curve using the intracranial tumor size as a determinant of cyst formation. The blue line represents the ROC curve from the patient cohort (AUC = 94.6%, standard error = 0.06, *p* value = 0.008, and 95% confidence intervals = 83.7–100%), while the red line is a reference line showing an AUC of 0.5.

Hyams tumor grade was reported for 25 patients (Figure 2), 4 patients had grade I tumors, 16 patients had grade II tumors, 4 patients had grade III tumors and 1 patient had a grade IV tumor. Twenty-eight patients were still alive (80.0%) at the time of analysis; the three patients that died all had intracranial extension (*p* = 0.0002, Fisher’s exact test) and higher-grade tumors (Hyams grade III or IV) (*p* = 0.0113, Fisher’s exact test). Higher tumor grade was significantly associated with the presence of intracranial extension (*p* = 0.0476, Fisher’s exact test). All four patients with evidence of cyst formation had Hyams grade II tumors, however there was no significant association between tumor grade and intracranial cyst formation (*p* = 0.5242, Fisher’s exact test). Additionally, there was no significant relationship between tumor grade and tumor size (*p* = 0.291, Mann–Whitney U test). The intracranial portion of tumors with cysts measured significantly larger than those without cysts (mean ± SD: 33.5 mm ± 10.6 versus 10.9 mm ± 7.8, *p* = 0.0003, independent-samples t-test), and in a binary logistics regression model, intracranial tumor size as measured in the craniocaudal dimension was a significant predictor of cyst formation (*p* = 0.002). A binary logistics regression plot of the data confirms the strong relationship between the response variable (tumor size) and the predictor variable (presence of cyst) (Figure 3A). A Receiver Operating Characteristic (ROC) curve demonstrated an area under the curve (AUC) of 94.6% with a standard error of 0.06, *p* value of 0.008, and 95% confidence intervals of 83.7–100% (Figure 3b).

## 4. Discussion

To our knowledge, this is the largest reported olfactory neuroblastoma patient cohort evaluating both imaging and corresponding pathology data. The sentinel publication concerning the topic of intracranial peritumoral cysts was that of Som et al. [3], which included 54 patients with sinonasal lesions with intracranial extension that were examined specifically for cysts along the intracranial margins of the lesions. However, no correlation with pathology, and specifically Hyams grading, was performed in this study. Several smaller studies have been published [6,7], such as the cohort of 11 patients evaluated for both MR imaging findings and staging (Kadish staging) by Yu et al., which found that 5 of 11 patients had intracranial extension and 4 of 11 patients had peritumoral cysts on MRI [8].

The results from our cohort suggest that cyst formation at the brain-tumor interface is an uncommon imaging finding in patients with olfactory neuroblastoma. We observed a significant relationship between higher tumor grade and the presence of intracranial extension; however, we did not observe a significant association between tumor grade and intracranial cyst formation or tumor grade and tumor size. Consistent with prior reports, survival was negatively impacted by the presence of intracranial tumor extension and a higher Hyams grade [9].

The extent of intracranial extension was by far the strongest predictor for peritumoral cyst formation in our cohort, as supported by both a binary logistics regression model and a ROC curve. The presence of peritumoral cysts has radiologically been described in other malignant sinonasal masses, such as sinonasal undifferentiated carcinoma [10] and sinonasal teratocarcinosarcoma [11], while both benign and malignant tumors of the nasal cavity can mimic olfactory neuroblastoma, including inverted papilloma, hemangioma, adenocarcinoma, squamous cell carcinoma and metastatic carcinoma [12,13]. Thus, our study suggests keeping olfactory neuroblastoma in a broad differential diagnosis for a sinonasal mass with intracranial extension without peritumoral cyst formation, and the utilization of a differential diagnosis for the imaging finding of peritumoral cysts rather than considering the presence of peritumoral cysts as pathognomonic for olfactory neuroblastoma.

The present study was not designed to evaluate the prevalence of cyst formation in other sinonasal malignancies; however, this could prove interesting in future work. The relationship between tumor size and apparent cyst formation at the brain–tumor interface suggests a possible mechanism of cerebrospinal fluid trapping as the cause of this radiological sign, regardless of histology, as the enlarging tumor displaces but does not yet invade the adjacent brain parenchyma. Some cases of peritumoral cysts in olfactory neuroblastoma do not show marginal enhancement, thus it is also possible that these cystic areas are degenerative in nature. As these lesions are not seen on pathology specimens nor reported in the pathology literature, it is unlikely that these findings represent true cysts. Thus, perhaps it is more accurate to refer to the findings on imaging as “peritumoral cystic spaces”.

The limitations of this study must be acknowledged, and include a somewhat small patient number. The low prevalence of cyst formation in this cohort limited our ability to perform some statistical comparisons, as did the relative low prevalence of higher-grade tumors. A limited number of imaging-derived features were evaluated in the present study. In future work, quantitative analysis of these tumors could be performed, using radiomics and/or artificial intelligence, to further evaluate the relationships examined in this study.

## 5. Conclusions

In the largest olfactory neuroblastoma patient cohort reported to date with both imaging and pathology data, cyst formation at the brain–tumor interface was an uncommon imaging finding, and tends to occur with a larger component of intracranial tumor extension. For the interpreting radiologist, our results suggest that the lack of a peritumoral cystic space should not preclude the inclusion of olfactory neuroblastoma in the differential diagnosis for nasal fossa lesions with intracranial extension, nor should the presence of a peritumoral cystic space for such a lesion preclude the inclusion of other pathologies.

## Figures and Tables

**Table 1:** Patient Characteristics.

Characteristic		All Patients (*n* = 31)
		
Gender	females	13 (41.9%)
	males	18 (58.1%)
Age (year)	median	55
	range	12–79
Intracranial extension	yes	18 (58.1%)
	no	13 (41.9%)
Cyst formation	yes	4 (22.2%)
	no	14 (77.8%)
Hyams grade	I/II	20 (80%)
	III/IV	5 (20%)

## Abbreviations

UPP&R HAND: UCLA patient-based Pathology & Radiology Head and Neck Database; MRI: magnetic resonance imaging; UCLA: University of California, Los Angeles.
